# Cone-Beam Computed Tomography for Temporomandibular Joint Imaging

**DOI:** 10.7759/cureus.31515

**Published:** 2022-11-14

**Authors:** Gunjan S Dhabale, Rahul R Bhowate

**Affiliations:** 1 Public Health Dentistry, Sharad Pawar Dental College & Hospital, Datta Meghe Institute of Medical Sciences, Wardha, IND; 2 Oral Medicine and Radiology, Sharad Pawar Dental College & Hospital, Datta Meghe Institute of Medical Sciences, Sawangi (Meghe), Wardha, IND

**Keywords:** cone-beam computed tomography, temporomandibular joint, temporomandibular disorder, mandibular asymmetry, osseous changes

## Abstract

The temporomandibular joint (TMJ) can be viewed using various imaging techniques. Due to relatively low radiation doses and excellent spatial resolution, cone-beam computed tomography (CBCT) is being utilized more frequently in dental-maxillofacial imaging. For the diagnosis and treatment of TMJ disorders, an imaging examination is required. The osseous compartment is visualized using conventional CT, and CBCT and soft tissue imaging are extremely well appreciated on MRI. However, conventional TMJ imaging has its limitations due to its two-dimensional view and adjacent anatomical superimposition. TMJ imaging helps analyze the cortical and the bony compartment's trabaculae and assess the degree of skeletal abnormalities. TMJ imaging protocols are also used to evaluate treatment responses.

CBCT is the three-dimensional imaging of the bony compartment and joint space and the morphology of the bone visualized by removing superimposition and distortion. Compared to multislice CT, CBCT produces high-resolution multiplanar images with a reduced dose of radiation. The role of CBCT imaging in determining the normal bony anatomy and pathological changes is appropriately delineated in this paper.

This work will focus on the use of CBCT for the examination of TMJ in various patient categories, including those with osteoarthritis, remodeling, ankylosis, trauma, rheumatoid arthritis, synovial chondromatosis, and other intracapsular pathologies.

## Introduction and background

The temporomandibular joint (TMJ) is the diarthrodial synovial joint with a disc, two bones, a fibrous capsule, intra-auricular fluid, and synovial stratum and ligaments. In conventional imaging, the bony components are adjacent to the TMJ. The base of the skull is superimposed on the joint compartment, resulting in indistinct images [1,2]. While high doses of radiation and long scanning time are important limitations of CT imaging, cone-beam computed tomography (CBCT) is the preferred imaging modality for the maxillomandibular region due to low doses of radiation, high spatial resolution, and less scanning time [2,3]. Similar to the collimated fan beam utilized in spiral CT, an X-ray beam shaped like a cone is utilized in CBCT, which is sometimes referred to as volumetric CT (VCT). In addition to up to six times lower radiation exposure, CBCT also offers a shorter scanning time compared to conventional CT scans [2].

In 1982, the Mayo Clinic was the first to utilize the prototype of a clinical CBCT scanner. Almost a decade after their first use for angiographic applications, commercial CBCT scanners were introduced [3].

**Figure 1:**
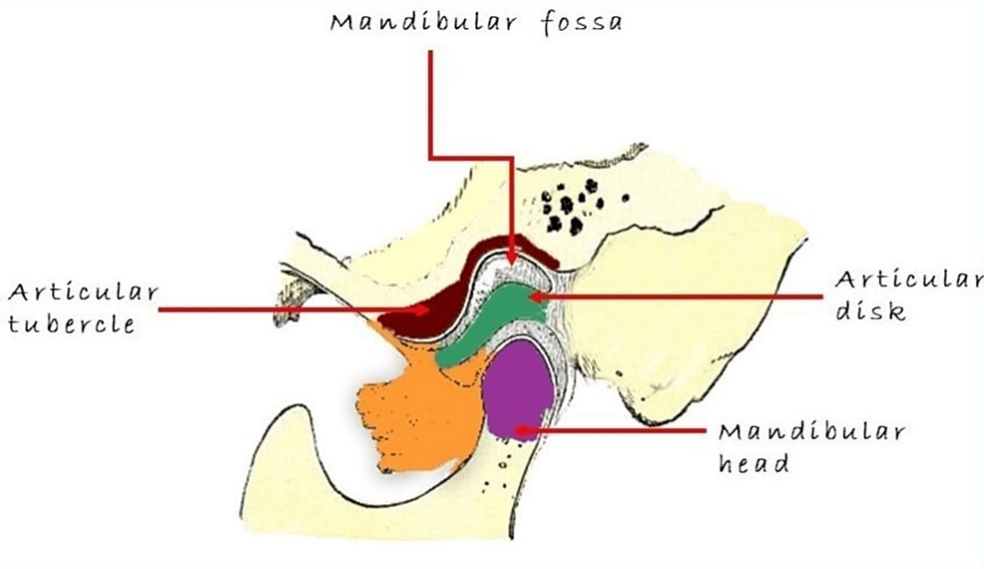
Anatomy of the temporomandibular joint.

Degenerative bony alterations include erosion, osteophytes, subchondral bone sclerosis, and pseudocysts, as well as flattening of the cortical outline [4,5]. There is abundant literature on CBCT, including reviews on the use of CBCT for TMJ imaging [6-9]. CBCT is a reliable technique for examining the TMJ's osseous components in coronal, axial, and sagittal planes [10] (Figure 1).

## Review

CBCT protocol for TMJ imaging

It is important to understand that the term CBCT is not related to other specific imaging methods. The field of view (FOV), type of detector, and detector voxel size are modifiable parameters for defining optimal images in diagnosis. In CBCT devices, the image of the FOV can be recorded in the dimensions 4 cm × 4 cm × 4 cm. A small FOV can be selected to cover the condylar head, articular eminence, and glenoid fossa of a single TMJ. This high-resolution scan helps delineate minute alterations in the TMJ's osseous structures [11]. The collimated image size ranges from 10 cm to 20 cm, with voxel sizes ranging from 200 µm up to 400 µm. Both TMJs may be included in the image volume depending on the FOV selected. Oral radiologists have to note that, as the voxel sizes and FOV increase, the image resolution decreases, which is responsible for poor osseous delineation [11]. Multiplanar pictures in the sagittal, transverse, and frontal planes in CBCT can be reconstructed in the image volume on these planes for easy TMJ visualization, and these reconstructed images help determine the condyle location within the glenoid fossa [11].

Appearance of bony TMJ defects on CBCT imaging

CBCT is used to assess the bony cortices of the head of the condyle and glenoid fossa, as well as articular eminence and its trabecular pattern, calcification level of the head of the condyle, and TMJ spaces. This visualization helps determine disease progression and treatment outcomes.

The following sections describe some of the common TMJ problems where CBCT can aid in diagnosis and treatment planning.

Remodeling/Age Changes in the Head of the Condyle

Remodeling is a natural procedure that tries to adjust the TMJ structure under the biomechanical forces of mastication to maintain occlusal relation and the homeostasis of the joint form and function. Masticatory forces may be responsible for the altered morphology of articular eminence and flattening of the head of the condyle, which leads to thickened articular eminence with sclerosis of the subchondral bone. Remodeling of the bony compartments of TMJ is asymptomatic most of the time but is considered abnormal when symptomatic, and subsequently, TMJ imaging is required.

Osteoarthritis

Osteoarthritis (OA) of the TMJ is a degenerative disease that affects women more than men, with a women-to-men ratio of 7:1. Biomechanical stress, attrition, internal joint derangement, and microtrauma induced by teeth grinding and chewing on one side, as well as the loss of molar support, are responsible for OA. In the case of OA, the degenerated fibrocartilage releases degraded photolytic enzymes into the synovial fluid, which is responsible for the secondary inflammatory response, causing joint components to degrade. The uneven bony cortex of the head of the condyle, erosion, osteophyte, and subchondral cyst are examples of bony changes caused by OA. Reduced joint space, subchondral sclerosis, and the flattening of the head of the condyle, glenoid fossa, and articular eminence are some of the other changes. These osseous changes in OA are best revealed in CBCT imaging compared to panoramic radiography, linear tomography, and MRI. CBCT imaging helps in disease staging and monitoring and depicting these changes caused by OA [12].

Ankylosis

The restricted movement of the mandible is a clinical symptom of TMJ ankylosis. TMJ ankylosis is either fibrous, fibro-osseous, or bony and is intracapsular rather than extracapsular in terms of location. The most common etiological factors are trauma, infections, rheumatoid arthritis (RA), post-surgical complications, and the fracture of the condyle. In the case of ankylosis, CBCT imaging has significant analytic and therapeutic advantages and helps assess the extent of bony unions. The imaging results reveal a partial or complete eradication of the TMJ joint space, osseous linking the condyle and the temporal bone, and zygomatic bone process, which is sometimes represented by an amorphous abnormal bone deposition [13,14]. In fibrous ankylosis, a variety of TMJ movements are possible with normal osseous components, and sometimes discrete calcifications and erosion of the head of the condyle and the remodeling of the condylar head occur [13,15].

Inflammatory Arthritis/Rheumatoid Arthritis

Systemic illnesses responsible for synovial membrane inflammation are RA, juvenile idiopathic arthritis, spondyloarthritis, ankylosing spondylitis, lupus erythematous, and Reiter's syndrome [16]. The most common inflammatory arthritis is RA. The most commonly affected periarticular structures are the synovial membrane capsule, tendon sheath, and fibrous ligaments.

When a TMJ inflammatory condition is suspected, CBCT is used to view minor osseous abnormalities. Cortical erosion is most commonly found on the articular eminence and anterior part of the head of the condyle. Subchondral sclerosis, flattening of articulating surfaces, subchondral cysts, and osteophyte production are also visible in CBCT imaging. Inflammatory arthritis has non-specific radiographic symptoms that are similar to OA [17].

Articular Disc Internal Derangements

Internal derangements of the TMJ are conditions where the articular disc is in an aberrant position with respect to the mandibular condyle and articular eminence. Displacement of the anterior part of the articular disc, with or without reduction, is a common symptom of this condition [18,19]. Internal derangements clinically present as clicking or a restricted opening of the afflicted joint. Importantly, in CBCT imaging, the articular disc cannot be seen. OA is typically caused by chronic internal derangements, showing cortical erosions. The observation of erosions in CBCT scans, along with clinical clicking and limited jaw movements, helps identify the disease condition. In such cases, MRI helps reveal the definitive position of the disc [19,20].

For example, in disc displacement, the posterior position of the head of the condyle in the glenoid fossa causes a reduction in posterior joint space. In the case of disc displacement without reduction, on the other hand, minimal translational motions of the condyle were reported. In these two situations, CBCT helps identify internal derangements [21].

Trauma

TMJ fractures are most common at the neck of the condyle and are frequently associated with displacement of the head of the condyle. The site of the fracture, either intravascular, extracapsular, or sub-condylar, its direction, and the presence and extent of the fracture are all visible in CBCT imaging [22]. A limited CBCT scan is enough to visualize localized fractures of the condyle, but a full FOV scan might be required to delineate the extent of fracture in the condylar head. For the entire traumatized maxillofacial region, multiplanar CBCT imaging is used to examine the skeleton.

Synovial Chondromatosis

Synovial chondromatosis (SC) is caused by synovial joint tissue chondrometaplasia. It typically influences the large joint and is infrequent in the TMJ [23]. Trauma, microtrauma, and degenerative arthritis have been suggested as the secondary causes of SC. Multiple loose calcified bodies within the joint space, along with the widening of the joint space, and sclerotic or irregular glenoid fossa are observed in CBCT images in the case of SC. Scans taken in the open-mouth position often reveal the shift in the location of these loose calcified bodies and OA, which typically causes concomitant osseous alterations.

Abnormalities During TMJ Development

TMJ developmental abnormalities, such as the aplasia of condyle, hypoplasia, and hyperplasia, can manifest as facial asymmetry. CBCT provides 3D information that can be used to determine the existence and severity of the maxilla and mandible asymmetry. A narrow FOV CBCT scan may be sufficient depending on the task of diagnosis [24]. A full FOV scan, on the other hand, may be required to assess mandibular development and asymmetry on both sides.

Coronoid Hyperplasia

Coronoid hyperplasia (CH), which causes an elongated coronoid process, is the unnoticed yet common cause of the reduction of mouth opening. During the opening of the mouth, the coronoid process impinges on the arch of the zygomatic bone or the zygomatic process of the maxilla, restricting jaw movement. A full FOV CBCT scan helps examine the magnitude of the coronoid process [25].

Directions for the use of CBCT imaging in the future

The osseous structures of the TMJ can be viewed clearly in CBCT imaging, and currently, the assessment of alterations in osseous morphology is based on qualitative observations. Recent studies have employed this image-processing method to ascertain whether these radiographic alterations can be measured and associated with other health outcomes. Shape correspondence, a 3D surface mapping technique, is employed to map the condylar anatomy and its changes due to OA [26].

These methods revealed the variance between asymptomatic condyles and OA. Estimating the magnitude of these anatomical alterations is crucial. Such imaging technologies can help examine temporal changes in joints and detect anatomical alterations that might have clinical implications for prognosis and management.

Discussion

Modality of CBCT in TMJ Imaging

Due to minimal radiation exposure to patients, its compact apparatus, and its capacity to produce multilane reformation and 3D images, CBCT has a distinct advantage over other imaging techniques [27]. For evaluating fractures, degenerative changes, erosions, infections, airway volume, sinus, nasal passage, and congenital defects, CBCT imaging is the best method [28]. Although CBCT is primarily a 3D imaging technique, it can also produce good-quality two-dimensional images. The type of image output is automatically set during the 360-degree rotation under the density of the tissue. Moreover, the total scan time in CBCT is only 76 seconds. Due to these reasons, CBCT is considered a "smart beam technology" [5]. CBCT imaging of the TMJ allows for the precise measurement of the surface and volume of the condyle, which is crucial for treating patients with TMD [5]. 

Table 1 shows the different studies conducted on CBCT of the TMJ. These studies are explored further in the discussion section.

**Table 1:** Studies conducted for TMJ imaging by using CBCT CBCT: cone-beam computed tomography; TMJ: temporomandibular joint; TOMO: tomography; MDCT: multidetector computed tomography; RTB: reversed twin block; PBMT: photobiomodulation therapy.

Author	Year	Type of study	Sample size	Result	Conclusion
Honey et al. [29]	2007	In-vitro cross-sectional study	37 TMJ articulations from 30 skulls	CBCT offered greater accuracy and reliability than linear TOMO and Panoramic projection.	CBCT images are more accurate and reliable for TMJ imaging.
Alexiou et al. [30]	2009	Research	71 TMJ photographs	Reduced joint spaces were found in 50% of the joints, and mandibular fossa sclerosis was found in 68% of joints.	Degenerative arthritis is associated with aging.
Zain-Alabdeen, Alsadhan [31]	2014	Research	10 TMJs from five dry human skulls	Intra-observer reliability was higher for MDCT than CBCT. Inter-observer reliability was higher for CBCT than for MDCT.	CBCT requires less radiation exposure for imaging TMJ.
Nah [32]	2012	Original article	440 TMJs from 220 patients	Sclerosis is the most frequent bony change in the condyle.	The criteria for osteoarthritis diagnosis need to be more thorough or specific.
Librizzi et al. [11]	2011	Research	16 TMJs containing natural and artificially created erosions and 16 normal TMJs	The diagnostic efficacy of the CBCT area under the curve for the 6-in Field of View was significantly greater than that of the 12-in Field of View.	CBCT scans with a small field of view are more effective than those with a large field of view.
Paknahad et al. [33]	2015	Research article	Mandibular vertical asymmetry in 20 patients with unilateral and 20 with bilateral cleft lip and palate and 20 patients with normal occlusion	There was no significant difference in mandibular asymmetry based on sex.	There is no significant difference in condylar position between symptomatic and asymptomatic groups.
Khwanda et al. [34]	2022	Randomized controlled clinical trial	40 children (12 females and 28 males) between 9 and 12 years with skeletal class III were assigned to the RTB+PBMT group or the control group (RTB only).	Condylar volume was significantly increased in the RTB group. The RTB group showed the most significant changes.	There were no considerable differences in condylar position after the class III treatment between the RTB and the RTB+PBMT groups. Only difference in the condylar volume was observed.

Studies on CBCT for TMJ Imaging

Honey et al. [29] examined a sample of 37 TMJ articulations from 30 skulls, with normal morphology of condyle (n = 19) or lateral pole erosion (n = 18), to detect the accuracy level of CBCT for TMJ imaging compared to panoramic radiology and linear tomography. This in-vitro, cross-sectional, observational study reported that CBCT is superior to tomography and panoramic projection in terms of reliability and accuracy. Thus, CBCT was deemed more reliable and accurate for TMJ imaging than any other imaging technique [29].

Alexiou et al. [30] collected computed data from 71 TMJ photographs of patients with degenerative arthritis to assess osteoarthritic changes. The parameters included were changes in the condyle's bony structure, joint spaces, and bony changes in the mandibular fossa. The authors reported reduced joint spaces in 50% of the joints studied and mandibular fossa sclerosis in 68% of the joints [30]. CBCT technique helped detect the defects in the TMJ.

Zain-Alabdeen and Alsadhan [31] retrieved data compiled through naked-eye inspection of 110 sites in 10 TMJs from five dry human skulls to assess the reliability and accuracy of CBCT compared to multidetector computed tomography (MDCT). The authors used sensitivity, specificity, and kappa static as the parameters for the analysis. Their findings indicated that (a) the sensitivity of both techniques was comparatively low; (b) specificities were high and comparable; (c) intraobserver reliability was higher for MDCT than for CBCT; and (d) interobserver reliability was higher for CBCT than for MDCT. However, CBCT required less radiation exposure to patients for the imaging of the TMJ with suspected osseous changes in the surface [31]. Thus, CBCT techniques are more sensitive and reliable for TMJ imaging than other techniques.

Nah [32] used CBCT data and the first-visit clinical records of 440 TMJs from 220 consecutive patients with TMD to observe the condylar bony changes in the patients. The author reported sclerosis as the most frequent bony change in the condyle (30.2%), followed by surface erosion (29.3%), articular surface flattening (25.5%), and deviations in forms, including cane-shaped, medial, or lateral pole depression of the TMJs, flattening of the posterior surface of the condyle, and the bifid-shaped condyle [32]. The bony changes in TMD patients (e.g., sclerosis) can easily be detected by CBCT.

Librizzi et al. [11] collected 32 samples of TMJs, of which 16 contained natural and artificially created erosions and 16 were normal TMJs, to investigate the effect of FOV and voxel size on the diagnostic efficacy of CBCT scan for detecting erosions in the TMJ. They used thermoluminescent dosimetry chips to calculate the absorbed dose and effective dose. Imaging protocols were compared using the receiver operating characteristics curve. Their findings indicated that the diagnostic efficacy of the CBCT area under the curve for 6-in FOV was significantly greater than that of 12-in FOV. The effective dose for 6-in FOV was 558 µSv, and that for 12-in FOV was 916µSv [11]. 

Paknahad et al. [33] observed the CBCT scans of three groups to differentiate the vertical asymmetry of the mandible among 20 patients with unilateral cleft lip and palate, 20 patients with bilateral cleft lip and palate, and 20 patients with normal occlusion. Their results indicated that there was no significant difference in mandibular asymmetry between these groups in terms of sex. All asymmetry indices were significantly higher in the unilateral cleft group than in the other two groups [33].

Khwanda et al. [34] evaluated the effect of photobiomodulation therapy (PBMT) on the TMJ components following class III treatment with the reversed twin block (RTB) appliance in growing patients. Their results indicated that condylar volume was significantly increased in the RTB group only by a mean of 287.97 mm cube. There were no considerable differences in the condylar position after the class III treatment between the RTB and RTB+PBMT groups [34]. 

## Conclusions

The use of CBCT to estimate the bony components of the TMJ is rapidly increasing. Compared to CT, CBCT produces high-resolution multiplanar images of the TMJ and requires lower radiation doses. CBCT provides crucial information for diagnosing a variety of TMD conditions, including OA, inflammatory arthritis, trauma, and developmental abnormalities. CBCT may be the modality of choice for determining the osseous morphology of the TMJ due to its high dimensional accuracy in measuring facial structures, including the TMJ. For the assessment of TMJs, CBCT quickly replaced CT as the more affordable and dose-effective alternative. In CBCT, the scan time is shorter and the radiation dose to patients is lower compared to conventional CT. CBCT generates images of excellent diagnostic quality. This novel technology is an extremely important diagnostic tool, and its popularity is gradually increasing.
